# Pseudomonas hefeiensis sp. nov., isolated from the rhizosphere of multiple cash crops in China

**DOI:** 10.1099/ijsem.0.006303

**Published:** 2024-03-27

**Authors:** Kaiji Liao, Qiang Li, Jun-Zhou Li, Hai-Lei Wei

**Affiliations:** 1 State Key Laboratory of Efficient Utilization of Arid and Semi-arid Arable Land in Northern China, Key Laboratory of Microbial Resources Collection and Preservation, Ministry of Agriculture and Rural Affairs, Institute of Agricultural Resources and Regional Planning, Chinese Academy of Agricultural Sciences, Beijing 100081, PR China; 2 College of Life Science and Technology of Huazhong Agricultural University, Wuhan 430070, PR China; 3 Shandong Tudacu Fertilizer Co. Ltd, Jining 272000, PR China

**Keywords:** crops rhizosphere, plant growth-promoting rhizobacteria, polyphasic taxonomy, *Pseudomonas*

## Abstract

Three bacterial strains, FP250^T^, FP821, and FP53, were isolated from the rhizosphere soil of oilseed rape, licorice, and habanero pepper in Anhui Province, Xinjiang Uygur Autonomous Region, and Jiangsu Province, PR China, respectively. All strains were shown to grow at 4–37 °C and pH 6.0–9.0, and in the presence of 0–4.0 % (w/v) NaCl. Phylogenetic analyses based on 16S rRNA gene sequences or housekeeping genes (16S rRNA, *gyrB*, *rpoB*, and *rpoD*) and phylogenomic analysis showed that strains FP250^T^, FP821, and FP53 belong to the genus *Pseudomonas*, and are closely related to *Pseudomonas kilonensis* DSM 13647^T^, *Pseudomonas brassicacearum* JCM 11938^T^, *Pseudomonas viciae* 11K1^T^, and *Pseudomonas thivervalensis* DSM 13194^T^. The DNA G+C content of strain FP205^T^ was 59.8 mol%. The average nucleotide identity and digital DNA–DNA hybridization values of strain FP205^T^ with the most closely related strain were 93.2 % and 51.4 %, respectively, which is well below the threshold for species differentiation. Strain FP205^T^ contained summed feature 3 (C_16 : 1_* ω*6*c* and/or C_16 : 1_* ω*7*c*), summed feature 8 (C_18 : 1_* ω*7*c* and/or C_18 : 1_* ω*6*c*) as major fatty acids, and diphosphatidylglycerol along with phosphatidylethanolamine and aminophospholipid as major polar lipids. The predominant isoprenoid quinone was ubiquinone-9. Based on these phenotypic, phylogenetic, and chemotaxonomic results, strain FP205^T^ represents a novel species of the genus *Pseudomonas*, for which the name *Pseudomonas hefeiensis* sp. nov. is proposed. The type strain is FP205^T^ (=ACCC 62447^T^=JCM 35687^T^).

## Introduction

Based on the List of Prokaryotic Names with Standing in Nomenclature (https://lpsn.dsmz.de/), there are currently approximately 330 species of *Pseudomonas* with validly published and correct names, and this number is growing quickly [1]. Based on the Genome Taxonomy Database (GTDB R214), the genus *Pseudomonas* has 7100 genomes containing isolated and uncultured micro-organism genomes [2]. Based on multilocus sequence analysis (MLSA) and whole genome sequencing analyses, *Pseudomonas* species are divided into 11–13 groups [3,5], among which the *Pseudomonas fluorescens* group is the largest and is subdivided into eight to nine subgroups, several of which are widely regarded as beneficial [3,5].

Organisms of the genus *Pseudomonas* are Gram-stain-negative, rod-shaped, motile with one or multiple polar ﬂagella, non-spore-forming, and obligate aerobes [6]. Members of the genus share a common fatty acid profile consisting of C_16 : 0_, summed feature 3 (C_16 : 1_* ω*7*c* and/or C_16 : 1_* ω*6*c*), and summed feature 8 (C_18 : 1_* ω*7*c* and/or C_18 : 1_* ω*6*c*). The cells contain ubiquinone-9 (Q-9) as the major respiratory quinone [7]. This genus is known for its wide-ranging metabolic diversity and has been identified in a range of environments such as soil, rivers, lakes, plants, animals, tar pits, glaciers, and diesel fuel tanks [8,9].

The rhizosphere is a well-studied biological niche in which plants recruit beneficial bacteria through root exudate secretion [10,11]. Through a variety of processes, including direct antagonistic interactions with pathogenic bacteria, the synthesis of compounds that stimulate plant development and beneficial micro-organisms help plants resist biotic and abiotic challenges [12,14]. These micro-organisms are collectively known as plant-growth-promoting rhizobacteria [15].

In the present study, we used polyphasic methods to identify three strains (FP205^T^, FP821, and FP53) isolated from plant rhizospheres [16]. We propose that FP205^T^, FP821 and FP53 represent a novel species of *Pseudomonas* named *Pseudomonas hefeiensis*.

## Isolation and ecology

We collected rhizosphere soil samples from oilseed rape (*Brassica napus*), licorice (*Glycyrrhiza uralensis*), and habanero pepper (*Capsicum chinense*) in Hefei (117° 16′ 94.26″ E 31° 72′ 42.83″ N), Anhui Province; Urumqi (87° 41′60.32″E, 43°47′70.85″N), Xinjiang Uygur Autonomous Region; and Yangzhou (119°19′ 14.69″ E 32° 27′ 80.91″ N), Jiangsu Province, PR China, respectively. For isolation, 1 g soil was suspended in 9 ml sterile ddH_2_O and shaken at 28 °C with 200 r.p.m. for 1 h. Serial dilutions of the suspensions in sterile ddH_2_O were spread on King’s B (KB) agar medium and incubated at 28 °C for 3 days. Single colonies were randomly picked, and purified three times using the same medium. Each pure culture was obtained and stored at −80 °C in 20 % aqueous glycerol suspension (v/v) for long-term preservation and further characterization.

## Phylogenetic and phylogenomic analyses

For phylogenetic analyses, the 16S rRNA gene sequences of the three strains were amplified using the bacterial universal primers 27F and 1492R and then sequenced using Sanger sequencing [17]. Near-full-length 16S rRNA gene sequences (1401 bp) were uploaded to EzBioCloud to obtain the closest relatives according to sequence similarity values [18]. Closely related strains were confirmed using the GTDB and TYGS servers [2,19]. Three isolates, 22 type species, and one undescribed species were used for subsequent analyses. To ascertain the phylogenetic position of the novel isolate, the 16S rRNA sequences were aligned using muscle [20]. A maximum-likelihood (ML) tree was reconstructed via mega X using the nearest-neighbour interchange branch-swapping algorithm and the K2P model with 1000 bootstrap replications [21]. MLSA was performed using four concatenated housekeeping genes (16S rRNA, *gyrB*, *rpoB*, and *rpoD*). An ML tree was also built using mega X after aligning the concatenated sequences using muscle. Bootstrap values were obtained using 1000 bootstrap replicates. For phylogenomic analyses, 92 bacterial core genes based on the Up-to-date Bacterial Core Gene (UBCG) tool were identified using default parameters, and an ML tree was reconstructed using the method described above [22].

**Fig. 1.**
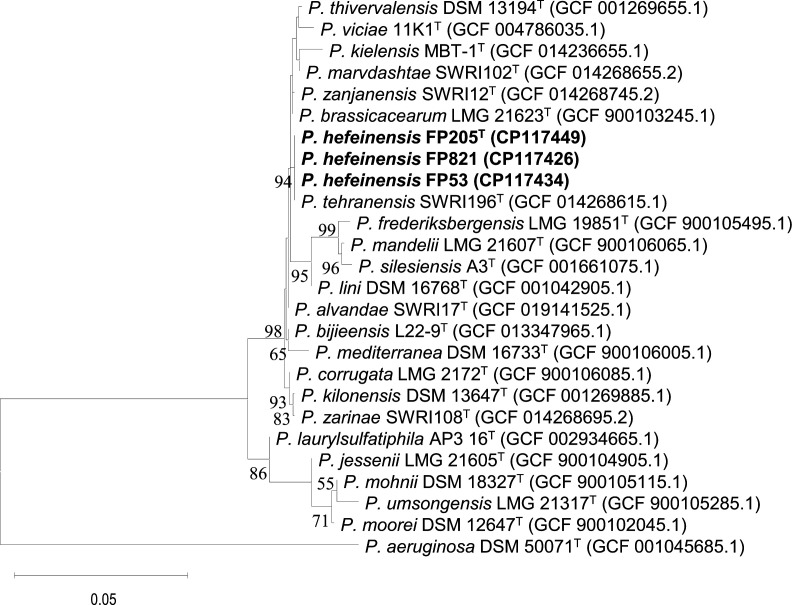
Maximum‐likelihood tree based on the nearly complete 16S rRNA gene sequences. The tree showed the relationships between the three isolates (FP205^T^, FP821, and FP53) and closely related type strains. The tree was reconstructed with mega X and rooted at the midpoint. Bootstrap values>50 % (based on 1000 repetitions) are shown at the branch nodes. The scale bar corresponds to 0.01 substitutions per nucleotide position. GenBank accession numbers of these genomes are given in parentheses.

**Fig. 2.**
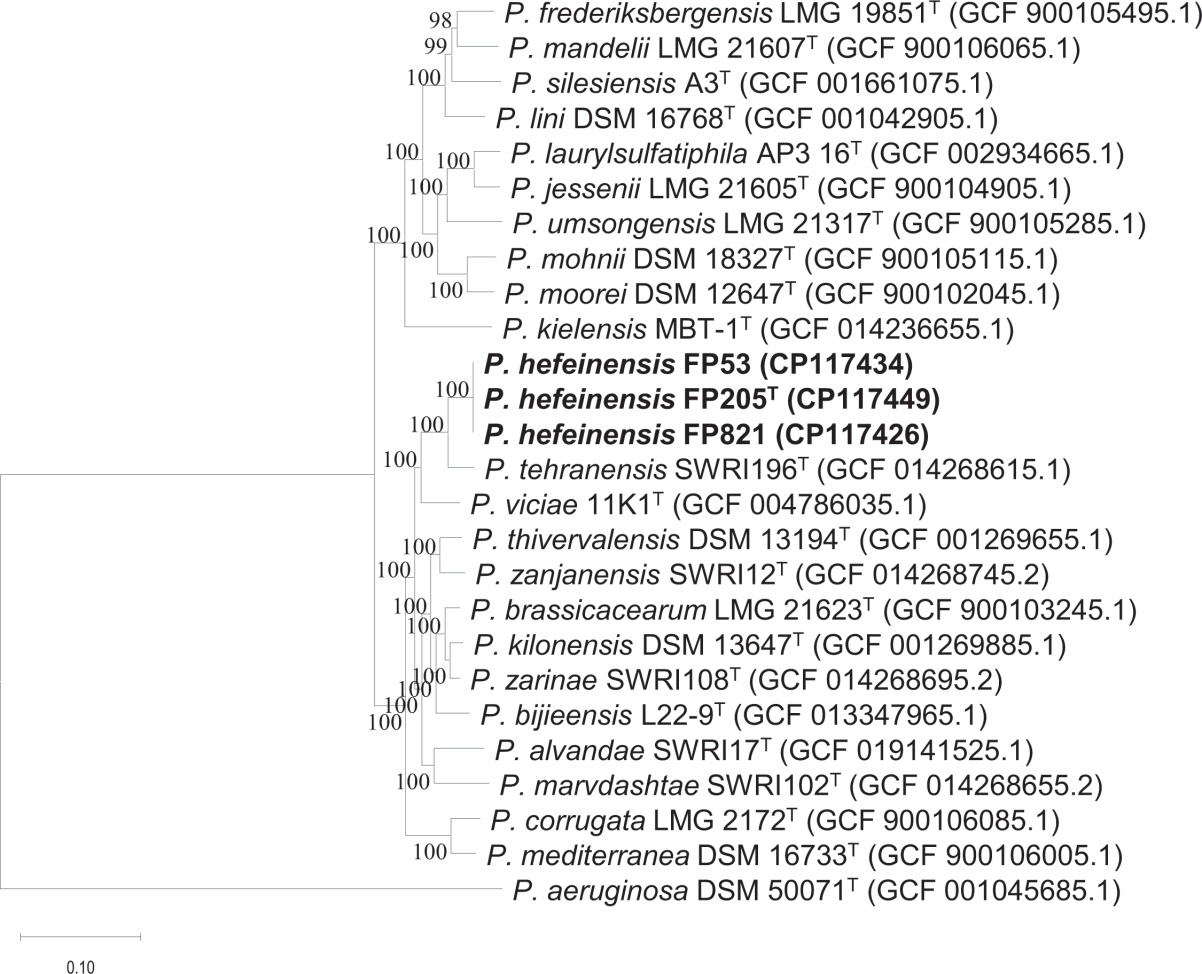
Maximum‐likelihood tree reconstructed using the concatenated alignment with 92 bacterial core genes. The tree represents the relationships between the three isolates (FP205^T^, FP821, and FP53) and closely related species. Phylogenomic inference was performed using the UBCG tool (concatenated alignment of 92 core genes) inferred using mega X. Numbers on the nodes represent the bootstrap values (based on 1000 replicates; values >50 % are shown). Scale bar, 0.05 substitutions per nucleotide. GenBank accession numbers of these genomes are provided in parentheses.

The 16S rRNA gene sequences of three isolates showed 99.9 % similarity to each other. In contrast, strain FP205^T^ was closely related to *Pseudomonas brassicacearum* JCM 11938^T^ (99.7 %, similarity), *Pseudomonas thivervalensis* DSM 13194^T^ (99.5 %, similarity), and *Pseudomonas kilonensis* DSM 13647^T^ (99.4 %, similarity). The 16S rRNA phylogenetic tree revealed that FP205^T^, FP821, FP53, and *Pseudomonas tehranensis* SWRI196^T^ clustered into a branch with bootstrap values of >50 % (Fig. 1). The MLSA and phylogenomic tree showed that FP205^T^, FP821, and FP53 formed an independent branch with 100 % confidence, which formed a sister group to *P. tehranensis* SWRI196^T^ with 100 % confidence (Figs 2 and S1, available in the online version of this article). The all trees showed that *P. heifeiensis* sp. nov. belonged to the *Pseudomonas corrugata* subgroup.

## Genomic and comparative analysis

The complete genomes of the strains FP205^T^, FP821, and FP53 were sequenced using the PacBio RSII and Illumina HiSeq platforms. Genomic DNA was extracted from each culture using a bacterial DNA kit (Omega), according to the manufacturer’s instructions. After quality determination using NanoDrop2000, genomic DNA libraries were prepared. HGAP (version 4.0) was used to assemble the *de novo* PacBio long reads [23], and Pilon (version 1.23) was used for error correction with Illumina short reads [24]. Genome assembly statistics were obtained with quast (version 5.0.2) [25], including the number of contigs, genome size, and genomic DNA G+C content. Prodigal was used to predict protein-coding sequences (CDS), and blast (version 2.14.0) [26] was used to search against the NCBI nr database. Non-coding RNA, primarily tRNA and rRNA, were predicted using RNA-scan-SE (version 1.4) [27] and RNAmmer (version 1.2) [28]. A genome-wide identification of the three strains was performed. The average nucleotide identity (ANI) and digital DNA–DNA hybridization (dDDH) values were analysed using fastANI (version 1.33) [29] and the Genome-to-Genome Distance Calculator (version 2.1) [30], respectively. AntiSMASH 7.0 was used to predict biosynthetic gene clusters (BGCs) with the detection strictness level set to 'relaxed' [31]. The genes for the carbohydrate active enzymes (CAZy) were identified based on the CAZy database [32]. MacSyFinder was used to identify secretion systems [33]. Some important genes that interact with plants were identified by blastp. Functional gene annotation was performed using blast against the Clusters of Orthologous Groups (COG) database [34].

The genomic characteristics of FP205^T^, FP821, FP53, and related type strains are presented in (Table S1). These results are consistent with those for the genus *Pseudomonas* [5]. Pairwise ANI and dDDH values were calculated to evaluate the genomic similarity among the three strains. ANI and dDDH values between any two of the three strains were 100 % and 99.9–100 %, respectively, indicating that they are the same species. However, the ANI values between three isolates and other closely related strains ranged 86.9–93.2 %, significantly lower than the 95–96 % threshold value recommended for species description [35]. Similarly, the dDDH values between isolates and other closely related type strains ranged from 32.7 to 51.4 %, which is below the 70 % threshold value for delineating species (Table 1) [36]. Both ANI and dDDH results, and phylogenetic analyses indicated that the three isolates represent a novel species within the genus *Pseudomonas*. The antiSMASH results showed that the genome of FP205^T^ had 12 putative BGCs that may be responsible for secondary metabolite production, with eight showing different levels of similarity to known BGCs (Table S2). Among them, there were clusters similar to those encoding fragin, fengcin, and lankaciden C [37,39]. These secondary metabolites have some antagonistic effect on micro-organisms. Several siderophores have been identified, including pyoverdine expressed by *Pseudomonas protegens* Pf-5 [40] and histicorrugatin [41]. Siderophores can help bacteria chelate iron in the environment, and thus achieve competitive colonization. Comparison of the distribution patterns of putative BGCs between FP205^T^ and its closest relatives revealed that the BGC of marinacarboline A was unique to that of FP205^T^. Marinacarboline is a *β*-carboline alkaloid and its pharmacological activity makes it desirable as a antiviral, antiparasitic or antimicrobial drug candidate (Table S3) [42]. Some genes or gene clusters involved in interactions with plants were detected in FP205^T^, including the synthesis of 1-aminocyclopropane-1-carboxylate deaminase, the production of indole-3-acetic acid, having type III and type VI secretion system, and a flagellar system (Table S4). FP205^T^ can produce a wide range of CAZymes, mainly including glycoside hydrolases and glycosyl transferases (Fig. S2). COG annotation assigned the 4944 CDSs of PF205^T^ to 23 categories. Among these, COG G (general function prediction only), COG E (amino acid transport and metabolism), and COG K(transcription) were the largest COG categories (Fig. S3). KEGG annotations revealed that 3729 CDSs of FP205^T^ were assigned to 42 pathway categories that harboured pathways for amino acid metabolism, carbohydrate metabolism, and cell motility.

**Table 1.** ANI and dDDH values (%) of FP205^T^, FP821, and FP53, with their most closely related species Data shown here have been sorted in order of their dDDH values against the FP205^T^ genome. The whole genome sequences used here are part of those used in the phylogenetic analyses.

Strain*	ANI (%)			dDDH (%)		
	FP205^T^	FP53	FP821	FP205^T^	FP53	FP821
FP205^T^	100	100	100	100	99.9	99.9
FP821	100	100	100	99.9	99.9	100
FP53	100	100	100	99.9	100	99.9
*Pseudomonas tehranensis* SWRI196^T^	93.2	93.2	93.2	51.4	51.4	51.4
*Pseudomonas viciae* 11K1^T^	88.8	88.8	88.8	36.1	36.1	36.1
*Pseudomonas zarinae* SWRI108^T^	88.9	88.9	88.9	36.0	36.0	36.0
*Pseudomonas brassicacearum* LMG 21623^T^	88.8	88.8	88.8	36.0	36.0	36.0
*Pseudomonas kilonensis* DSM 13647^T^	88.8	88.8	88.8	35.7	35.7	35.7
*Pseudomonas bijieensis* L22-9^T^	88.4	88.4	88.4	35.0	34.9	34.9
*Pseudomonas thivervalensis* DSM 13194^T^	88.2	88.2	88.2	34.4	34.4	34.5
*Pseudomonas zanjanensis* SWRI12^T^	88.0	88.0	88.0	34.0	34.0	34.0
*Pseudomonas marvdashtae* SWRI102^T^	87.0	86.9	87.0	32.7	32.7	32.7

## Physiology and chemotaxonomic characterization

Phenotypic and physiological characteristics of the three strains were assessed using metabolic and physiological tests after 24–48 h of aerobic culture in tryptic soy agar (TSA) medium at 28 °C. Colony morphology was examined after incubation for 24 h under optimal growth conditions. Cell morphology was observed using transmission electron microscopy (TEM). To identify the optimal growth conditions, we cultured each of the three strains under different pH values (pH 4–10 in 1 pH unit increment) adjusted using the buffer system described by Xu *et al*. (pH 4.0–5.0, 0.1 M sodium acetate–0.1 M acetic acid; pH 6.0–8.0, 0.1 M KH_2_PO_4_–0.1 M NaOH; pH 9.0–10.0, 0.1 M NaHCO_3_–0.1 M Na_2_CO_3_; pH 11.0, 0.05 M Na_2_HPO_4_–0.1 M NaOH) [43], different temperatures (4, 10, 20, 25, 30, 37, 40, and 45 °C), and different NaCl concentrations [0–5.0 % (w/v), 1.0 % increments] in TSA medium. Gram staining was performed using a Gram staining kit (bioMérieux) according to the manufacturer’s protocol. Fluorescent colonies were detected under a UV illuminator at 254 nm on KB agar [44]. The API 20NE system (bioMérieux) and GENIII MicroPlates (Biolog) were used to obtain detailed biochemical profiles of the three strains according to the manufacturer’s instructions. For chemotaxonomic analyses, including polar lipid, cellular fatty acid, and isoprenoid quinone compositions, cell biomass samples of FP205^T^ and reference strains incubated on TSA plates at 28 °C for 48 h were used. Polar lipids were extracted and analysed by two-dimensional thin-layer chromatography [45]. Well-grown cells were harvested and prepared according to standard protocols to identify cellular fatty acid profiles [46]. Cellular fatty acid profiles were determined using the Sherlock Microbial Identification System (midi) version 6.2 and the RTSBA 6 database. Respiratory quinones were extracted and analysed according to a previously described method and confirmed by high-performance liquid chromatography [47].

**Fig. 3.**
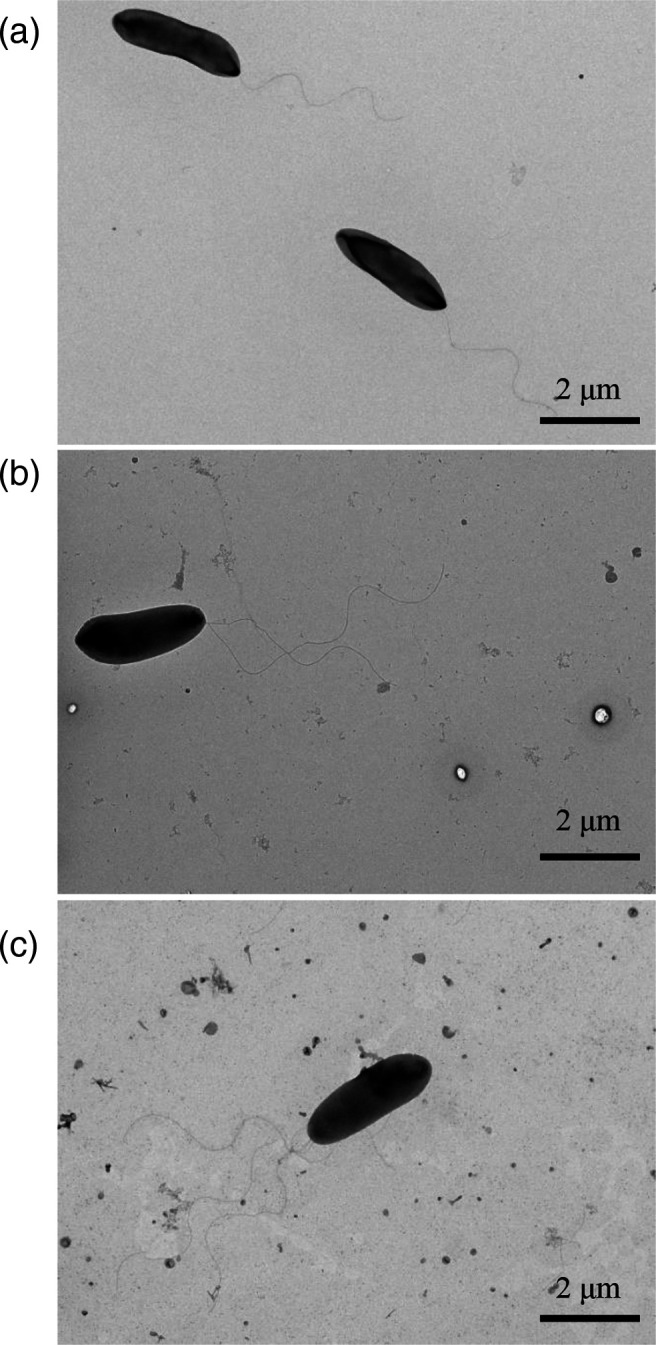
TEM of cells depicting the cell morphologies of the three strains. (**a**) FP205^T^, (**b**) FP821, and (**c**) FP53. Scale bar, 2 µm.

The colonies of FP205^T^, FP821, and FP53 were circular with entire margin, slightly convex, smooth, and pale yellow. Cells of three strains were Gram-stain-negative, rod-shaped, approximately 1.0×2.5 µm in size, and had one to multiple flagella (Fig. 3). The cells produced a fluorescent pigment in KB medium. Comparison of the biochemical and physiological characteristics of strain FP205^T^ and its closely related strains based on API 20NE data and Biolog GEN III MicroPlate resulted in differential characteristics. Their main different biochemical activities and physiological features are summarized in Table 2. Strains FP205^T^ and FP821 can grow at different temperatures between 4–37 °C with an optimum growth temperature of 28 °C. The pH range for growth was pH 6–9, with an optimum of pH 7. Salt tolerance was also assessed up to 4 % NaCl (w/v), with optimum growth with 0–1 % NaCl on TSA. API 20NE and Biolog GEN III analyses showed similarities and differences in carbon source utilization and chemosensitivity between FP205^T^ and closely related species (Table 2).

**Table 2.** Differential characteristics of strain FP205^T^, strain FP821, and most closely related strains of the genus *Pseudomonas* Strains: 1–1, FP205^T^; 1–2, FP821; 2, *P. viciae* 11K1^T^; 3, *P. kilonensis* DSM 13647^T^; 4, *P. brassicacearum* JCM 11938^T^; and 5, *P. thivervalensis* DSM 13194^T^. To build this table, data for FP205^T^, *P. kilonensis* DSM 13647^T^, *P. brassicacearum* JCM 11938^T^, and *P. thivervalensis* DSM 13194^T^ were obtained from the present study, whereas data for *P. viciae* 11K1^T^ were extracted from the literature [48]. +, Positive; −, negative; w, weak; na, not available.

Characteristic	1–1	1–2	2	3	4	5
**Growth range**						
Temperature (°C)	4–37	4–37	4–37	na	na	na
pH	6–9	6–9	6–10	na	na	na
NaCl (%, w/v)	0–4	0–4	0–5	na	na	na
**API 20 NE assay**						
Gelatin	+	+	−	−	+	+
*N*-Acetyl glucosamine	−	−	−	+	+	+
Gluconate	+	+	w	+	+	+
**Biolog GEN III assay**						
d-Fucose	−	−	w	−	w	+
d-Sorbitol	−	−	+	−	−	−
d-Mannitol	−	−	+	−	−	−
d-Serine	−	−	−	−	+	+
Guanidine HCl	w	w	w	+	w	+
d-Galacturonic acid	+	+	w	−	−	−
d-Glucuronic acid	+	+	w	−	−	+
Glucuronamide	+	+	w	−	w	+
Mucic acid	+	+	+	+	−	w
Bromo-succinic acid	+	+	w	−	−	−
Lithium chloride	+	+	w	−	−	−
*γ*-Amino-butryric acid	−	−	+	+	w	−

The cellular fatty acid profiles of strains FP205^T^ and FP821 were generally similar to those of the closely related type strains, except that the relative proportion of summed feature 3 of FP821 was lower than that in most other strains (Table 3). The major cellular fatty acids were summed feature 3 (C_16 : 1_* ω*6*c* and/or C_16 : 1_* ω*7*c*), summed feature 8 (C_18 : 1_* ω*7*c* and/or C_18 : 1_* ω*6*c*), C_12 : 0_, and C_16 : 0_. The hydroxylated fatty acids were C_10 : 0_ 3-OH, C_12 : 0_ 2-OH, and C_12 : 0_ 3-OH. The cyclopropane acid was C_17 : 0_ cyclo. FP205^T^ contained Q9 as the major respiratory quinone, which is present in most *Pseudomonas* species, such as *Peudomonas viciae* 11K1^T^ (Fig. S4) [48]. The major polar lipids in strain FP205^T^ were diphosphatidylglycerol, phosphatidylethanolamine, aminophospholipids, and unknown polar lipids (Fig. S5). The presence of diphosphatidylglycerol and phosphatidylethanolamine in strain FP205^T^ agrees with the closely related strain *P. viciae* KACC 21650^T^ [48] and other *Pseudomonas* species [49,51].

**Table 3.** Fatty acid composition (%) of strain FP205^T^, strain FP821, and most closely related strains of the genus *Pseudomonas* Strains: 1–1, FP205^T^; 1–2, FP821; 2, *P. viciae* 11K1^T^; 3, *P. kilonensis* DSM 13647^T^; 4, *P. brassicacearum* JCM 11938^T^; and 5, *P. thivervalensis* DSM 13194^T^.

Fatty acid	1–1	1–2	2	3	4	5
Summed features*						
3	22.4	12.0	22.4	34.2	23.6	19.6
8	15.7	12.1	19.5	15.0	12.3	11.1
**Saturated**						
C_12 : 0_	9.0	9.4	3.3	9.7	4.3	4.1
C_16 : 0_	24.2	23.0.	23.4	25.5	25.8	27.7
**Hydroxy**						
C_10 : 0_ 3-OH	3.5	6.2	3.8	4.1	1.0	4.0
C_12 : 0_ 2-OH	1.0	1.0	4.7	1.7	5.2	4.7
C_12 : 0_ 3-OH	4.4	5.3	4.7	4.4	5.0	5.0
**Cyclo**						
C_17 : 0_ cyclo	16.5	23.4	11.8	3.7	17.0	19.4

**Summed Ffeatures are fatty acids that cannot be resolved reliably from another fatty acid using the chromatographic conditions chosen. The MIDmidi system groups these fatty acids together as one feature with a single percentage of the total. Summed feature 3 was compriseds of C_16 : 1 _*ω*7*c*/C_16 : 1 _*ω*6*c* and/or C_16 : 1 _*ω*6*c*/C_16 : 1 _*ω*7*c*, and Ssummed feature 88 was compriseds of C_18 : 1 _*ω*7*c* and/or C_18 : 1 _*ω*6*c*.

## Description of *Pseudomonas hefeiensis* sp. nov.

*Pseudomonas hefeiensis* (he.fei.en’sis. N.L. fem. adj. *hefeiensis,* pertaining to Hefei, from which the strain was isolated).

Cells are Gram-stain-negative, rod-shaped, fluorescent, and approximately 1.0×2.5 µm. Growth on TSA occurs at 4–37 °C (optimum, 28 °C), at pH 6.0–9.0 (optimum, pH 7.0), and with 0–4 % NaCl (w/v; optimum, 0–1 %).

In the API 20NE test, positive responses are observed for gelatin and aesculin hydrolysis, 4-nitroso-*β*-d-methyl galactose, assimilation of l-arabinose, d-mannose, d-mannitol, gluconate, decanoic acid, malic acid, citric acid, and l-glutamic acid. In the Biolog GEN III system, positive for the utilization of *p*-hydroxy-phenylacetic acid, d-galacturonic acid, l-galactonic acid lactone, l-alanine, d-galactose, d-gluconic acid, d-glucuronic acid, citric acid, glucuronamide, d-fructose-6- phosphate, mucic acid, l-malic acid, and bromo-succinic acid, and weakly positive for the utilization of Tween 40, d-mannose, l-histidine, quinic acid, and formic acid. Cells are sensitive to 1 % sodium lactate, troleandomycin, lincomycin, vancomycin, aztreonam, fusidic acid, rifamycin SV, tetrazolium violet, lithium chloride, sodium butyrate, niaproof 4, tetrazolium blue, potassium tellurite, and guanidine HCl (weakly). The major cellular fatty acids are summed feature 3 (C_16 : 1_* ω*6*c* and/or C_16 : 1_* ω*7*c*), summed feature 8 (C_18 : 1_* ω*7*c* and/or C_18 : 1_* ω*6*c*), C_12 : 0_ and C_16 : 0_. The major quinone is ubiquinone (Q9). The major polar lipids are diphosphatidylglycerol, phosphatidylethanolamine, and aminophospholipids.

The type strain, FP205^T^ (=ACCC 62447^T^=JCM 35687^T^), was isolated from the rhizosphere of oilseed rape (*Brassica napus*) in Hefei, Anhui Province, PR China. GenBank accession numbers for the 16S rRNA gene and genome sequences are OR214998 and CP117449, respectively.

## supplementary material

10.1099/ijsem.0.006303 Uncited Supplementary Material 1.
