# Supplementary material for: Pseudomonas hefeiensis sp. nov., isolated from the rhizosphere of multiple cash crops in China
Source: Int J Syst Evol Microbiol. 2024 Mar 27;74(3):006303. doi: 10.1099/ijsem.0.006303 (PMC10995727; doi:10.1099/ijsem.0.006303)
Supplement: Uncited Supplementary Material 1. [file ijsem-74-06303-s001.pdf]

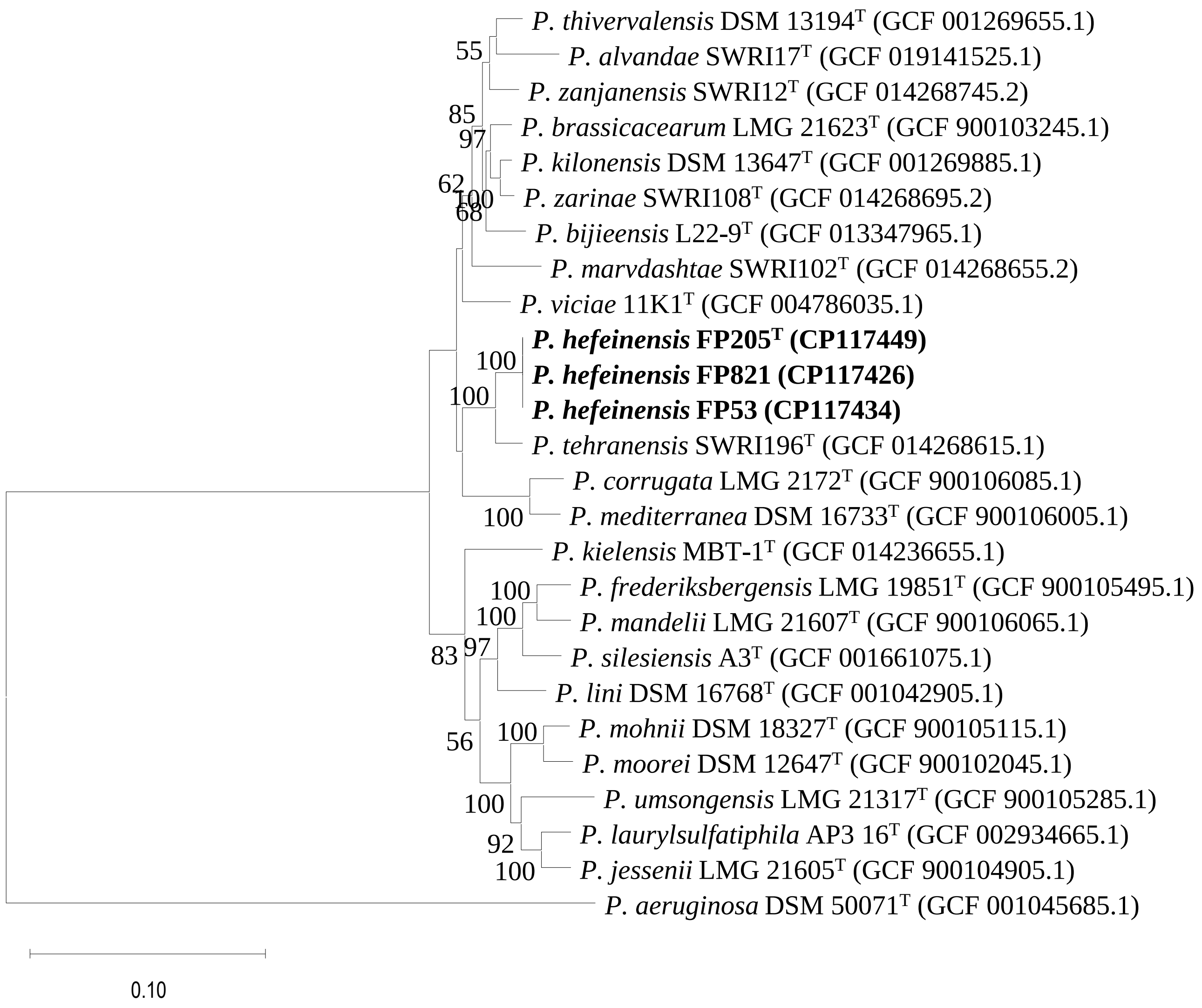

Fig. S1. Maximum-likelihood tree based on four housekeeping genes (16S rRNA, *gryB*, *rpoB, and rpoD*). The tree represents the relationships between the three isolates (FP205^{T}, FP821, and FP53) and closely related species. The tree was reconstructed using MEGA X and rooted at the midpoint. Numbers on the nodes represent the bootstrap values (based on 1000 repetitions; values > 50% are shown). Scale bar, 0.05 substitutions per nucleotide. GenBank accession numbers of these genomes are provided in parentheses.

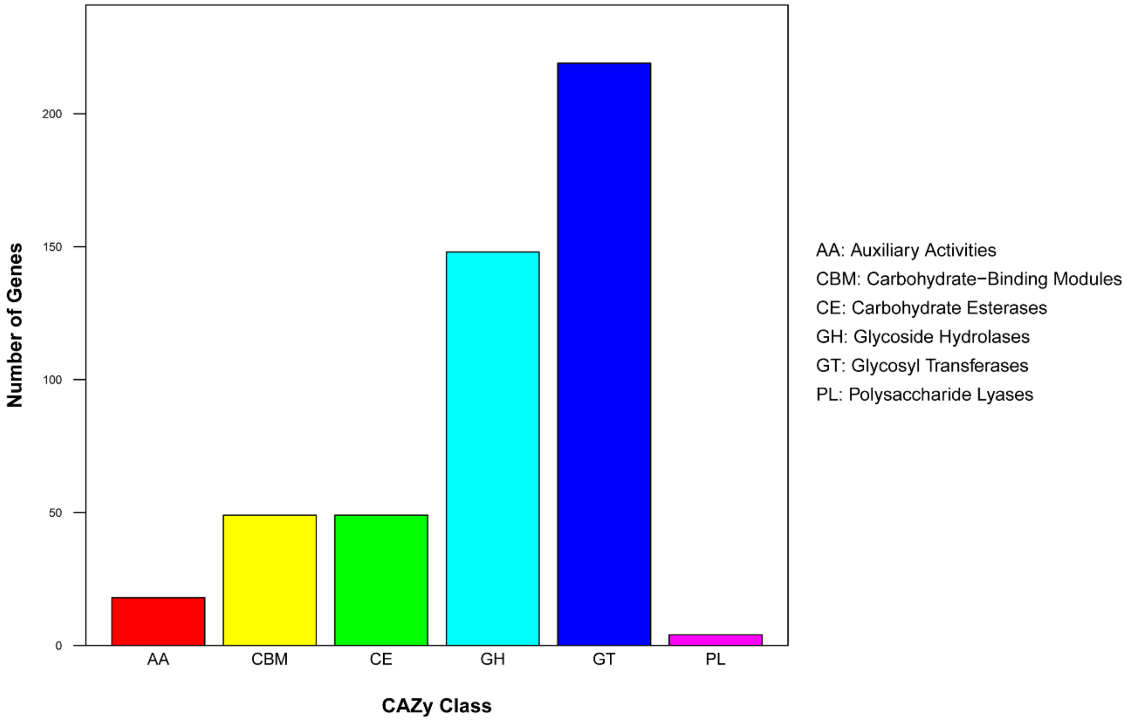

Fig. S2. Carbohydrate-hydrolyzing enzymes in FP205^{T}. Different colors indicate different classes.

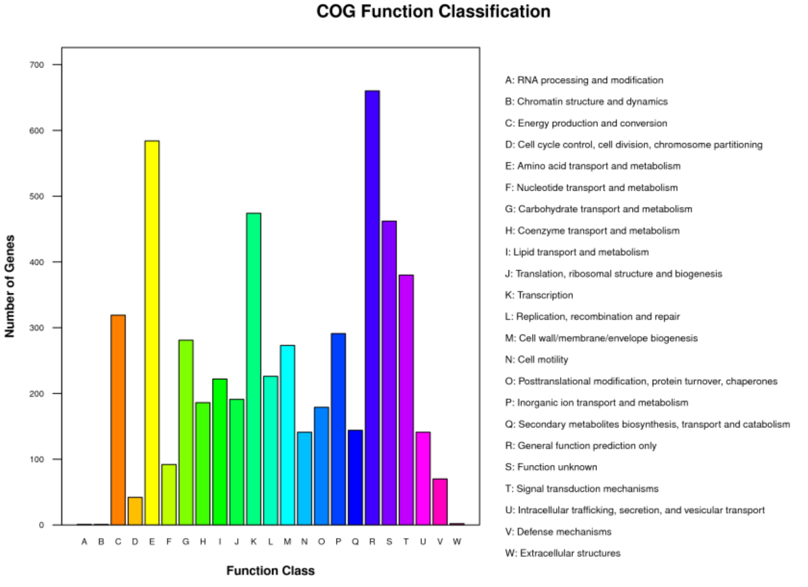

Fig. S3. COG annotation. Different colors indicate different functional categories.

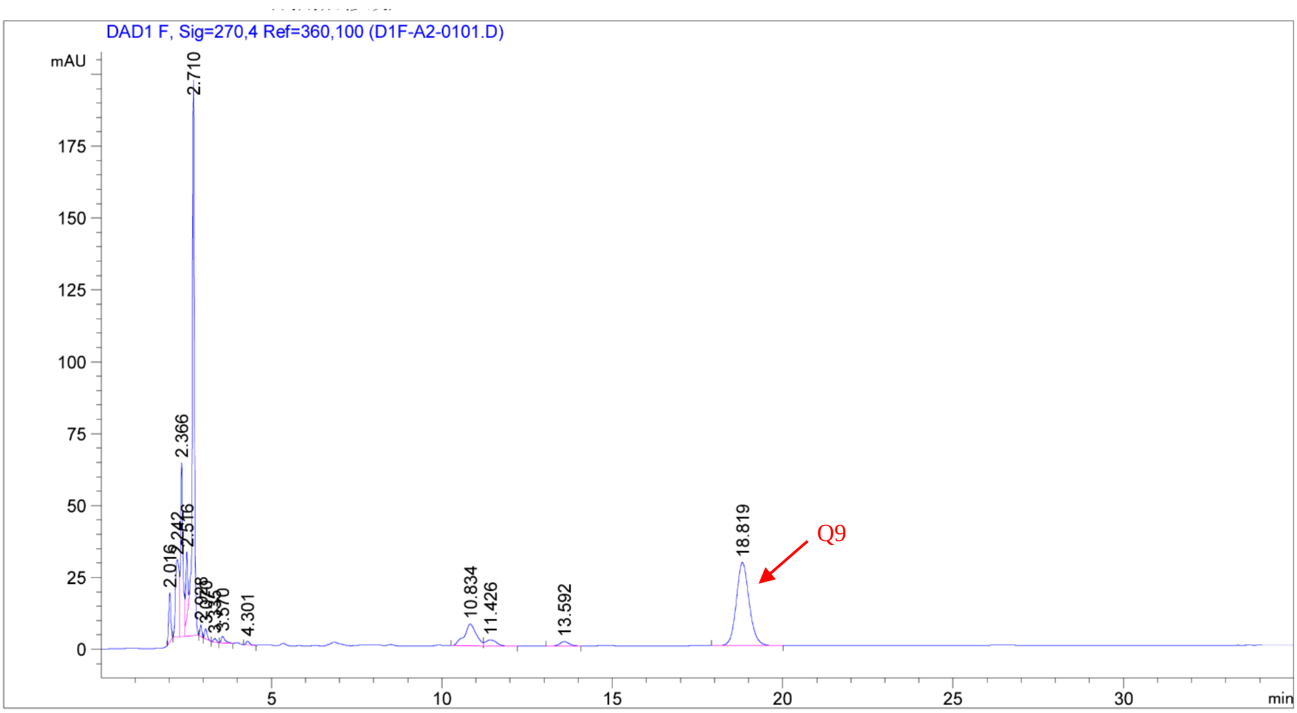

Fig. S4. Respiratory quinone for FP205^{T}. The red arrow indicates the retention time of the respiratory ubiquinone 9.

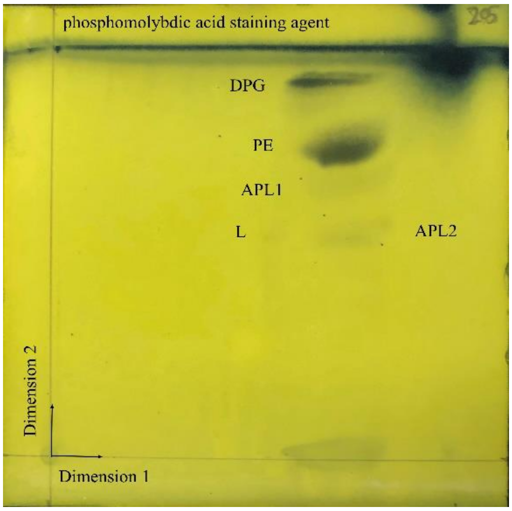

Fig. S5. Two-dimensional TLC plate of polar lipids extracted from FP205^{T} . The plate was sprayed with 10% (v/v) molybdophosphoric aicd to show all polar lipids present. PE, phosphatidylethanolamine; DPG, diphosphatidylglycerol; APL, aminophospholipid; L,unknown polar lipids.

Table S1. Genomic features of three isolates (FP205^T, FP821, and FP53) and closely related species.

Strains: 1–1, FP205^T; 1–2, FP821; 1–3, FP53; 2, *P. tehranensis* SWRI196^T, 3, *P. viciae* 11K1^T, 4, *P. kilonensis* DSM 13647^T; and 5, *P. brassicacearum* LMG 21623^T.

Genome feature	1–1	1–2	1–3	2	3	4	5
Genome size (Mbp)	6.5	6.5	6.5	6.0	6.7	6.4	6.8
Number of contigs	1	1	1	531	2	2	1
G + C content (%)	59.8	59.8	59.8	60.5	60.3	60.8	60.8
Protein coding genes (CDS)	5842	5884	5846	5349	5784	5592	5944
tRNA genes	69	69	68	52	66	70	67
rRNA genes	16	16	16	4	16	20	16

Table S2. Putative BGCs in the FP205^T genome predicted using antiSMASH.

Region	Cluster type	From	To	Information on most similar known cluster		
				Product	Similarity	MIBiG accession
1	NRPS-like	226202	264024	fragin	37	BGC0001168
2	Arylpolyene	522332	565943	APE Vf	40	BGC0002341
3	NRPS	1931691	2023537	Pf-5 pyoverdine	19	BGC0002688
4	Betalactone	2199366	2222617	fengcin	13	BGC0000131
5	NRPS	2612040	2688840	histicorrugatin	84	BGC0001133
6	Thioamitides	3090798	3132660	marinacarboline A	15	BGC0002560
7	RiPP-like	3655274	3663689	-	-	BGC0000556
8	NRPS	4402875	4455861	Pf-5 pyoverdine	10	BGC0000913
9	NAGGN	4600032	4614922	-	-	BGC0000846
10	RiPP-like	5011348	5022217	-	-	BGC0001579
11	Lanthipeptide-class-ii	5474272	5496596	-	-	BGC0000525
12	Redox-cofactor	5816726	5838891	lankaciden C	13	BGC0000906

Table S3. Type and number of putative BGCs in the genomes of FP205^T, FP53, FP821, and their phylogenomic neighbors predicted using antiSMASH.

Strains: 1–1, FP205^T; 1–2, FP821; 1–3, FP53; 2, *P. tehranensis* SWRI196^T, 3, *P. viciae* 11K1^T, 4, *P. kilonensis* DSM 13647^T; and 5, *P. brassicacearum* LMG 21623^T.

**Cluster type**	**Number of putative biosynthetic gene clusters**						
	**1–1**	**1–2**	**1–3**	**2**	**3**	**4**	**5**
fragin	1	1	1	1	1		1
APE Vf	1	1	1	1	1		1
Pf-5 pyoverdine	2	2	2	2	2	1	3
fengcin	1	1	1	1	1	1	1
histicorrugatin	1	1	1	2			1
marinacarboline A	1	1	1				
RiPP-like	2	2	2	2	1	2	1
NAGGN	1	1	1		1	1	1
lanthipeptide-class-ii	1	1	1		1	1	
lankaciden C	1	1	1	1	1	1	1
2,4-diacetylpholoroglucinol						1	1
butyrolactone						1	1
cepacin A						1	
pyochelin						1	
cepaciachelin					1	1	
pyoverdine SMX-1						1	
thanafactin A					1		
syringomycin					1		
hserlactone				1	1		

Table S4. Genes interacting with plants in the FP205^{T} genome

Function	Gene name	Gene id	Function	Gene name	Gene id
Flagellum	*flgB*	WLH14046.1	IAA	*iaaH*	WLH13597.1
	flgC	WLH14047.1		*iaaM*	WLH15308.1
	flgV	WLH11284.1	IAA degredation	*iacE*	WLH12808.1
	flgU	WLH11285.1		*IacC*	WLH12336.1
	flgT	WLH11286.1		*iacA*	WLH10185.1
	flgS	WLH11287.1		*IacG*	WLH10601.1
	flgR	WLH11288.1		*IacH*	WLH13597.1
	flgQ	WLH15532.1		*IacF*	WLH13678.1
	flgN	WLH11296.1	2,3-btd biosynthesis	*budC*	WLH12808.1
	flgJ	WLH11299.1		*ilvB*	WLH12971.1
	fliE	WLH11300.1		*ilvN*	WLH11772.1
T1SS	*omf*	WLH13279.1	Acetoin catabolism	*acoR*	WLH12841.1
	abc	WLH13007.1		*acoC*	WLH13255.1
	mfp	WLH13008.1		*acoA*	WLH14187.1
T3SS	*sctJ*	WLH12475.1		*acoB*	WLH10448.1
	sctC	WLH12480.1	Lipase	*LipA*	WLH13355.1
	sctU	WLH12485.1		*LipB*	WLH10119.1
	sctT	WLH12486.1	PAA degradation	*PaaD*	WLH13057.1
	sctS	WLH12487.1		*paaH*	WLH13067.1
	sctR	WLH12488.1		*PaaM*	WLH13088.1
	sctQ	WLH12489.1		*PaaL*	WLH13249.1
	sctN	WLH15577.1		*PaaE*	WLH13678.1
	sctV	WLH12493.1		*paaB*	WLH14306.1
T4P	*pilM*	WLH13201.1		*paaF*	WLH14578.1
	pilN	WLH13202.1		*paaK*	WLH14823.1
	pilO	WLH13203.1	PQQ biosynthesis	*pqqE*	WLH14273.1
	pilP	WLH13204.1		*pqqF*	WLH12288.1
	pilQ	WLH13205.1		*pqqB*	WLH12289.1
	pilAE	WLH11956.1		*pqqC*	WLH12290.1
	pilB	WLH11957.1		*pqqD*	WLH12291.1
	pilC	WLH11958.1	Spermidine	*speH*	WLH13106.1
	pilD	WLH11959.1		*speE*	WLH15273.1
	pilT	WLH12426.1	Metalloprotease	*aprA*	WLH10114.1
Tad	*tadV*	WLH14223.1	ACC deaminase	*acdS*	WLH15456.1
	tadZ	WLH14228.1			
	tadA	WLH14229.1			
	tadB	WLH14230.1			
	tadC	WLH14231.1			
	rcpA	WLH14234.1			
T6SS	*tssM*	WLH12668.1			
	tssL	WLH12955.1			
	tssK	WLH12669.1			
	tssJ	WLH12670.1			
	tssB	WLH12673.1			
	tssC	WLH12674.1			
	tssD	WLH12675.1			
	tssE	WLH12678.1			
	tssF	WLH12679.1			
	tssG	WLH12680.1			
	tssH	WLH12681.1			
	tssI	WLH12682.1			

*****IAA, indole3-acetic acid; PAA, phenylacetic acid; 2,3-btd, 2,3-butanediol; PQQ, pyrroloquinoline quinone; ACC, 1-aminocyclopropane-1-carboxylate
